# Effects of type of tropical pasture and concentrate supplementation level on the carcass traits of grazing lambs

**DOI:** 10.5194/aab-63-283-2020

**Published:** 2020-08-28

**Authors:** Gustavo Daniel Vega Britez, Fernando Miranda Vargas Junior, Marciana Retore, Marcelo Corrêa Silva, Luana Liz Medina Ledesma, Adrielly Lais Alves Silva, Jéssica Oliveira Monteschio, Tatiane Fernandes

**Affiliations:** 1 Facultad de Ciencias Agrarias, Universidad Nacional de Asunción Filial Pedro Juan Caballero, Pedro Juan Caballero, 79900-000, Paraguay; 2 Faculdade de Ciências Agrárias, Federal University of Grande Dourados (UFGD), Dourados, MS, 79804-970, Brazil; 3 Embrapa Agropecuária Oeste, Dourados, MS, 79804-970, Brazil; 4 Departamento de Zootecnia, Federal University of Roraima, Boa Vista, RR, 69310-000, Brazil

## Abstract

The nutritional requirements can be met, and carcass quality can be achieved by using concentrate supplementation in the diets of grazing lambs. This study evaluated the effects of different concentrate supplementation rates (0 %, 1.5 %, and 3 % of body weight) and tropical pastures (*Panicum maximum* cv. `Aruana' and *Brachiaria brizantha* cv. `Marandu') on lamb carcass traits. Thirty-six male Suffolk lambs, with an initial body weight of 22.54 ± 2.72 kg, were evaluated in a 3 × 2 factorial experimental design. The concentrate used consisted of milled soybean, maize, and oat grains. The pasture species affected empty body weight and commercial cuts. The use of concentrate supplementation affected carcass weight, yield, indexes, and commercial cuts. Also, the use of concentrate supplementation improved the weight of muscle and fat content. Based on discriminant analysis it is possible to identify the rearing systems, when all variables where used, or the level of concentrate supplementation when variables of weight were used. Carcasses of animals on grass-only diets were different and easier to discriminate. The use of concentrate supplementation on rearing lambs improves the quality of carcass traits. The period of finishing can be shorted with supplementation of 3 % of body weigh in Aruana and Marandu grass. The discriminant analysis can identify the differences between rearing systems based on all carcass traits. This analysis can be used to develop carcass traceability systems.

## 1 Introduction

Pastures are commonly used for meat production (Botinestean et al., 2016; Hamill and Botineştean, 2016). Because of the seasonality of tropical regions, the quality and availability of tropical pastures for livestock production differ over time (Araujo et al., 2018). This affects the nutritional supply to livestock and, consequently, carcass quality and price (Poppi and McLennan, 1995; Ephrem et al., 2015). Some of these limiting points can be overcome by using concentrate supplementation in the diets of the animals (Chestnutt, 1994; Jacques et al., 2011; Montossi et al., 2013), thereby achieving greater quality and productivity in the finished carcass and higher carcass yields (Murphy et al., 1994).

In general, the results of adding concentrate supplementation to the diet of animals depend on the forage offered. Some tropical forages are relatively more adapted to the tropical climate, such as *Urochloa* (syn. *Brachiaria*) *brizantha* cv. `Marandu' (Jank et al., 2014). While other tropical forage species have a greater nutritional value but, however, low the dry matter production, such as *Panicum maximum* cv. `Aruana'; those are desirable characteristics for the diet of grazing sheep (De Souza et al., 2014). Some studies have reported the effects of feed supplementation on the feeding behaviour and the uniformity of the performance of lambs raised on Aruana grass (Fajardo et al., 2015; Silveira et al., 2015), while other works have evaluated supplementation schemes on animal performance and production costs using Marandu grass (Geron et al., 2012; Carvalho et al., 2015). Also, some previous studies with Tifton indicate the use of concentrate can accelerate growth, improve the carcass yield, and increase fat content in the carcass (Carvalho et al., 2007; Fernandes et al., 2011). However, evaluations on lamb carcass yield and quality considering concentrate supplementation rates and grazing systems, based on Marandu or Aruana grass, are scarce. These studies may, however, be interesting for several segments of the production chain (Carrasco et al., 2009a; Montossi et al., 2013; Asadollahi et al., 2017). In this context, the objective of this study was to evaluate the effects of type of tropical pasture and concentrate supplementation level on the carcass traits of grazing lambs and determine which carcass traits can be used to identify the rearing finishing system.

## 2 Material and methods

All protocols and experimental procedures were approved by the Ethics Committee on Animal Experimentation (CEUA; protocol no. 027/2012) of the Federal University of Grande Dourados (UFGD), Brazil.

### 2.1 Description of the study area

The experiment was conducted in Ponta Porã, MS, Brazil, (22${}^{\circ }$32${}^{\prime }$56${}^{\prime \prime }$ S, 55${}^{\circ }$38${}^{\prime }$56${}^{\prime \prime }$ W, 642 ma.s.l.). According to the classification of Köppen, the region has a temperate climate (Cwa), with dry winters and rainy summers. Precipitation was 874 mm measured on the spot, with temperatures ranging from 18.6 to 29.0 ${}^{\circ }C$. The experiment was carried out from 9 December 2014 to 14 April 2015.

### 2.2 Animals and feed treatments

Thirty-six weaned Suffolk uncastrated male lambs were used in this study. All lambs belonged to the same herd of 150 matrices and were kept in a grazing system (Aruana grass) with breastfeeding and creep feeding supplementation (mixed of corn grain, soybean meal, and oat) until they were screened for the trial. Lambs with uniform weight (22.54 ± 2.72 kg of body weight) and age (90 d) were weaned to participate in the experiment.

The treatments consisted of three concentrate supplementation rates (0.0 %, 1.5 %, and 3.0 % of the body weight) and two tropical grass species (*Panicum maximum* cv. `Aruana' and *Brachiaria brizantha* cv. `Marandu'), with two replications of paddock and three animals in each paddock, a total of 36 animals for measurement and 12 experimental units (paddock).

The animals were labelled with ear tags and divided into 12 paddocks of 32 m × 32 m with continuous grazing. There was no control of the lambs' stocking rate. The biomass average weights of the fresh leaves ($\mathrm{kg}\;{\mathrm{ha}}^{-1}$) during the experiment were 1435.47 (0.0 %), 1455.17 (1.5 %), and 1620.23 (3.0 %) for Aruana grass and 2894.51 (0.0 %), 2857.67 (1.5 %), and 3180.28 (3.0 %) for Marandu grass, estimated via the visual technique (Haydock and Shaw, 1975). The average heights of the Aruana and Marandu grasses during the experimental period were 32.01 ± 7.45 and 51.16 ± 12.35 cm, respectively.

The concentrate used consisted of milled soybean, maize, and oat grains (Table 1), supplied daily in the mornings at 08:00 LT (local time). The supplement intake of the animals was adjusted by weighing the animals every 14 d at 08:00 LT, using an electronic balance with precision of 0.1 kg. The animals had access to water and mineral salt ad libitum throughout the experimental period.

**Table 1:** Proportion of ingredients in concentrate (milled mixture of grains) and chemical content of concentrate and forages.

	Concentrate	Aruana	Marandu
Ingredients (% of dry matter)			
Oat (grain)	45	–	–
Soya (grain)	33	–	–
Maize (grain)	22	–	–
Nutrient	Chemical content (mean and standard deviation)		
Dry matter (DM), % as feed	87.12	28.12 ± 0.84	31.50 ± 0.96
Crude protein, % of DM	21.84	16.58 ± 3.85	5.17 ± 1.36
Neutral detergent fibre, % of DM	35.44	62.94 ± 4.37	65.27 ± 6.23
Acid detergent fibre, % of DM	8.29	29.85 ± 4.24	30.95 ± 5.21
Ether extract, % of DM	8.83	1.32 ± 0.59	1.18 ± 0.32
Ash, % of DM	5.25	7.76 ± 0.84	8.48 ± 1.03

Worm infections were controlled by deworming the animals before the experiment and by monitoring through parasitological faecal examinations (Gordon and Whitlock, 1939) every 21 d, using as reference for deworming the limit 500 of eggs per gram of feces (EPG). Shade screens (70 %) of 3 m × 2 m were installed in each paddock.

### 2.3 Chemical analysis

Pasture samples were collected using a grazing simulation technique (Euclides et al., 1992), and concentrate samples were collected every 28 d and analysed to determine the chemical composition. All samples were dried at 60 ${}^{\circ }C$ for 72 h and grounded; to determine dry matter they were dried at 105 ${}^{\circ }C$ for 24 h; the crude protein content was evaluated using the Dumas procedure (AOAC, 2005). Neutral detergent fibre (NDF) and acid detergent fibre (ADF) were evaluated according method described by Van Soest et al. (1991). The ethereal extract (EE) content was evaluated by the acid hydrolysis method (AOAC, 2005). The ash content was evaluated gravimetrically by incineration of the samples in a muffle oven at 550 ${}^{\circ }C$ for 2 h. The metabolisable energy (EM) of forage and of the concentrated supplement was calculated based on the equations described in the NRC (2007).

### 2.4 Slaughter procedure, carcass measurements

The lambs, independent of the treatment, were slaughtered according to the body condition score (BCS) 2.5–3.0 (scale 1–5), according to Russel et al. (1969), or at the age of 6 months, resulting in slaughtering at 73, 77, 91, 98, 105, and 126 experimental days, with six animals each day. The animals were slaughtered at 110 km from the experimental site, in an experimental abattoir from Federal University of Grande Dourados.

The lambs were weighed (body weight at slaughter – BWS) after solid fasting of approximately 16 h and slaughtered according to the procedures described in the Regulations for Industrial and Sanitary Inspection of Animal Origin Products (Brazil, 2000). Subsequently, their skins were removed, and the gastrointestinal organs were emptied to measure the empty body weight (EBW = BWS - gastrointestinal content). The carcasses were weighed to obtain hot carcass weight (HCW) and hot carcass yield (HCY = HCW / BWS × 100) and subsequently placed in a refrigerator at 4 ${}^{\circ }C$ for 24 h, hung by the gastrocnemius tendons. The cold carcass weight (CCW), including kidneys and pelvic fat, was then measured and used to calculate the cold carcass yield, CCY = (CCW / BWS) × 100, and cooling losses, CL = (CCW - BWS) × 100 / CCW. Subsequently, a symmetrical longitudinal section on the middle of the carcass was performed with an electric saw to visually evaluate the fat condition (FC) of the carcass according to the methodology of Bligh and Dyer (1959). A cross section in the longissimus thoracis et lumborum muscle of the left half of the carcass, between the 12th and 13th ribs, was carried out for subjective evaluations of meat texture (TEX), marbling (MBL), and surface colour (COL), as described previously Bligh and Dyer (1959).

Carcass leg length (LL), rump width (RW), internal length (ICL), carcass compacity index (CCI = CCW / ICL), and leg compacity index (LCI = RW / LL) were measured according to Colomber-Rocher et al. (1988). The leg muscle index (LMI) was calculated using the equation proposed by Purchas et al. (1991).

### 2.5 Cuts and dissection

The commercial cuts were evaluated on the left half of the carcass, and the proportion of tissue components was evaluated by dissection of the leg. The left half of the carcass was divided into five anatomical regions (Colomber-Rocher et al., 1988), namely leg, palette, rib, loin, and neck, and each region was weighed individually to determine their yields (%) to the cold half-carcass weight. The tissue components of the right leg (muscle, bone, fat, and remainder parts) were evaluated by dissection (Colomber-Rocher et al., 1988), by weighing individually on a semi-analytical balance. The total muscle content (TMC; kg), total fat content (TFC; kg), total bone content (TBC; kg), and their yields (%) to the cold half-carcass weight, in addition to the ratios muscle-to-bone (M / B) and muscle-to-fat (M / F), were calculated to evaluate the carcass composition.

Subcutaneous fat thickness (SFT; mm) was measured in the longissimus thoracis et lumborum muscle on the surface of the 13th rib, at 11 cm from the lumbar back line, using a digital calliper.

### 2.6 Experimental design and statistical analysis

A completely randomised design was used, with a 3 × 2 factorial scheme (two pasture species and three concentrate supplementation rates) and six lambs per treatment. Analysis of variance was performed using the PROC GLM in SAS (Statistical Analysis System, version 9.2). The statistical model used was $Y_{ij}=\mu +P_{i}+C_{j}+(P\times C{)}_{jj}+e_{ij}$, where μ is the overall mean, $P_{i}$ is the effect of pasture (iAruana, Marandu), $C_{j}$ is the effect of concentrate supplementation (j=0.0 %, 1.5 %, and 3.0 %), (P × C) is the interaction effect of grass and supplementation,and $e_{ij}$ is the experimental error, with fixed (concentrate supplementation rates and pasture species) and random (slaughter day) effects. Differences were considered at a 5 % significance level, according to the least squares means with adjustment for Tukey's test.

**Table 2:** Different subsets of variables established to develop multivariate analysis and evaluate the discrimination between carcasses of male Suffolk lambs fed with different concentrate supplementation and tropical grass species.

Subsets of measurements	N	Variables
Weight in kilograms	16	Final body weight; body weight at slaughter; empty body weight; hot carcass weight; cold carcass weight; total muscle content; total fat content; subcutaneous fat; intramuscular fat; total bone content; total neglected${}^{a}$ content; leg weight; palette weight; rib weight; loin weight; neck weight
Percentage	11	Hot carcass yield; cold carcass yield; leg weight; palette weight; rib weight; loin weight; neck weight; total muscle content; total fat content; total bone content; total neglected content
Subjective and objective	8	Leg muscularity index; carcass compacity index; leg compacity index; fat condition; texture; colour; marbling; body score
Indicators of adipose tissue	6	Cover fat thickness; subcutaneous fat thickness; fat condition; total fat content${}^{b}$; subcutaneous fat${}^{b}$; intramuscular fat${}^{b}$
Indexes of muscularity and compacity	3	Leg muscularity index; carcass compacity index; leg compacity index

N: number of variables in each subset. ${}^{a}$ Non-muscle, non-subcutaneous fat, and non-bone content. ${}^{b}$ Measured in kilograms.

A joint analysis of variables of the carcass traits was carried out to evaluate the discriminant capacity of treatments (concentrate supplementation rates and pasture species); these analyse were performed using the DISCRIM procedure from SAS (Statistical Analysis System, version 9.2). The 29 evaluated variables were sorted into five subsets of carcass traits, according to an arbitrary criterion of grouping based on quantitative variables (Table 2). Explanatory procedures, using different subsets of variables, were carried out to compensate for similar numbers of characteristic variables (n=29) compared to the numbers of carcasses (n=36), which could imply in an unstable linear discriminant function and lead to bias.

## 3 Results

The concentrate supplementation used significantly affected all measures of carcass weight (kg) and yield (%; Table 3). Lambs with supplementation had a higher final body weight, BWS, EBW, HCW, CCW, HCY, CCY, and LMI, without differences between 1.5 % and 3.0 % supplementation. For CL, the animals with 1.5 % of concentrate supplementation had lower losses than those without supplementation, with no difference for 3.0 % of supplementation. Concentrate supplementation affected the SFT and CCI (Table 3). However, there were no differences between 1.5 % or 3.0 % levels of concentrate supplementation (Table 3). For finishing period, we observed an interaction between concentrate supplementation and tropical pasture. Lambs supplemented with 3 % had shorter time (days) between weaning and slaughter in both grass pastures(Aruana and Marandu). For animals fed with Aruana grass, there were no differences between without and with 1.5 % of supplementation (105 to 91 d, respectively) and animals on Marandu grass with 1.5 % of supplementation (98 d). However, animals fed with Marandu grass without concentrate supplementation had longer finishing period (126 d without supplementation).

**Table 3:** Means, standard deviation, and effect of concentrate supplementation (C), tropical pasture type (P), and interaction (C × P) related to the carcass weight, yield, and indexes of Suffolk lambs.

	Concentre supplementation			Tropical pasture		p value		
	0 %	1.5 %	3 %	Aruana	Marandu	C	P	C × P
FBW, kg	30.50 ± 5.58${}^{b}$	38.95 ± 3.87${}^{a}$	36.72 ± 4.35${}^{a}$	36.67 ± 4.38	34.10 ± 6.34	0.02	0.16	0.24
BWS, kg	27.25 ± 5.17${}^{b}$	35.64 ± 3.55${}^{a}$	33.17 ± 4.26${}^{a}$	33.16 ± 4.41	30.87 ± 5.96	0.01	0.19	0.31
EBW, kg	21.25 ± 4.49${}^{b}$	29.99 ± 3.16${}^{a}$	28.50 ± 3.42${}^{a}$	28.02 ± 4.13	25.14 ± 5.32	< 0.01	0.08	0.24
HCW, kg	11.42 ± 1.83${}^{b}$	16.65 ± 2.16${}^{a}$	15.43 ± 2.53${}^{a}$	15.24 ± 2.80	13.76 ± 3.44	0.01	0.15	0.35
CCW, kg	10.82 ± 2.68${}^{b}$	16.08 ± 2.19${}^{a}$	14.96 ± 2.41${}^{a}$	14.66 ± 2.70	13.25 ± 3.47	0.01	0.15	030
Hot carcass yield, %	41.35 ± 3.23${}^{b}$	46.61 ± 2.34${}^{a}$	46.34 ± 3.10${}^{a}$	45.70 ± 3.08	43.84 ± 3.93	0.02	0.18	0.36
Cold carcass yield, %	39.14 ± 3.30${}^{b}$	45.00 ± 2.59${}^{a}$	44.98 ± 2.91${}^{a}$	43.98 ± 3.15	42.11 ± 4.29	< 0.01	0.20	0.31
Cooling losses, %	10.87 ± 2.01${}^{a}$	8.44 ± 1.77${}^{b}$	9.72 ± 2.28${}^{\mathrm{ab}}$	9.67 ± 2.23	9.68 ± 2.27	0.01	0.49	0.12
SFT, mm	0.75 ± 0.35${}^{b}$	1.75 ± 0.63${}^{a}$	1.61 ± 0.67${}^{a}$	1.41 ± 0.64	1.33 ± 0.78	0.04	0.76	0.91
LMI, $\mathrm{kg}\;{\mathrm{cm}}^{-1}$	0.40 ± 0.03${}^{b}$	0.44 ± 0.04${}^{a}$	0.43 ± 0.03${}^{a}$	0.43 ± 0.04	0.42 ± 0.04	0.02	0.16	0.32
CCI, $\mathrm{kg}\;{\mathrm{cm}}^{-1}$	0.19 ± 0.04${}^{b}$	0.26 ± 0.03${}^{a}$	0.25 ± 0.03${}^{a}$	0.24 ± 0.03	0.23 ± 0.05	< 0.01	0.15	0.16
BCS at slaughter	1.67 ± 0.16	2.63 ± 0.14	2.75 ± 0.12	2.41 ± 0.12	2.29 ± 0.18	0.09	0.32	0.87
Finishing period, d	115.5 ± 0.33${}^{c}$	94.5 ± 0.27${}^{b}$	76.0 ± 0.27${}^{a}$	90.33 ± 0.22${}^{a}$	100.33 ± 0.26${}^{b}$	< 0.01	< 0.01	< 0.01

Different lower-case letters in the same line indicate significant differences (p<0.05) between concentrate supplementation. Different upper-case letters in the same line indicate significant differences (p<0.05) between tropical pasture. FBW: final body weight; BWS: body weight at slaughter; EBW: empty body weight; HCW: hot carcass weight; CCW: cold carcass weight; SFT: subcutaneous fat thickness; LMI: leg muscularity index; CCI: carcass compacity index; finishing period: days between weaning and slaughter; BCS: body condition score (1–5 scale).

Concentrate supplementation improved the weight (kg) of commercial cuts (Table 4). Considering the percentage variables, no effects of supplementation were observed (Table 4). The TMC and TFC (in kg) were higher with concentrate supplementation, with no differences between supplementation level; however, the muscle proportions, when expressed as a percentage, were not affected by concentrate supplementation (Table 5). Supplementation resulted in higher TFC values in percent, and lower TBC values in percent, with no differences between the two supplementation levels for both (Table 5). Concentrate supplementation resulted in a low muscle–fat ratio but did not affect the muscle–bone ratio (Table 5).

**Table 4:** Means, standard deviation, and effect of concentrate supplementation (C), tropical pasture type (P), and interaction (C × P) related to commercial cuts of the left half of the carcass of Suffolk lambs.

	Concentre supplementation			Tropical pasture		p value		
	0 %	1.5 %	3 %	Aruana	Marandu	C	P	C × P
Leg weight, kg	1.91 ± 0.46${}^{b}$	2.69 ± 0.39${}^{a}$	2.56 ± 0.41${}^{a}$	2.50 ± 0.42	2.27 ± 0.59	0.02	0.18	0.28
Palette weight, kg	1.12 ± 0.30${}^{b}$	1.58 ± 0.24${}^{a}$	1.46 ± 0.27${}^{a}$	1.45 ± 0.26	1.32 ± 0.36	0.03	0.22	0.36
Rib weight, kg	1.28 ± 0.32${}^{b}$	2.03 ± 0.33${}^{a}$	1.81 ± 0.28${}^{a}$	1.78 ± 0.37	1.63 ± 0.46	0.01	0.17	0.48
Loin weight, kg	0.58 ± 0.17${}^{b}$	0.93 ± 0.16${}^{a}$	0.84 ± 0.19${}^{a}$	0.84 ± 0.19	0.72 ± 0.24	0.02	0.11	0.31
Neck weight, kg	0.38 ± 0.09${}^{b}$	0.54 ± 0.09${}^{a}$	0.52 ± 0.12${}^{a}$	0.52 ± 0.11${}^{A}$	0.44 ± 0.12${}^{B}$	0.02	0.07	0.33
Leg weight, %	36.15 ± 1.28${}^{a}$	4.63 ± 1.04${}^{b}$	35.61 ± 1.54${}^{\text{ab}}$	35.25 ± 1.18	35.68 ± 1.64	0.12	0.45	0.56
Palette weight, %	21.29 ± 0.70	20.31 ± 1.19	20.24 ± 0.98	20.51 ± 0.95	20.72 ± 1.23	0.14	0.63	0.85
Rib weight, %	24.31 ± 0.96	26.10 ± 1.96	25.18 ± 1.42	25.01 ± 1.48	25.39 ± 1.89	0.19	0.91	0.48
Loin weight, %	10.90 ± 0.94	11.95 ± 0.92	11.65 ± 1.22	11.86 ± 1.02	11.14 ± 1.08	0.16	0.11	0.35
Neck weight, %	7.32 ± 0.86	6.97 ± 0.94	7.29 ± 0.92	7.35 ± 0.68	7.04 ± 1.09	0.43	0.74	0.71

Different lower-case letters in the same line indicate significant differences (p<0.05) between concentrate supplementation. Different upper-case letters in the same line indicate significant differences (p<0.05) between tropical pasture.

**Table 5:** Means, standard deviation, and effect of concentrate supplementation (C), tropical pasture type (P), and interaction (C × P) related to composition and muscularity of the leg from the carcass of Suffolk lambs.

	Concentre supplementation			Tropical pasture		p value		
	0 %	1.5 %	3 %	Aruana	Marandu	C	P	C × P
Total muscle content, kg	0.78 ± 0.21${}^{b}$	1.07 ± 0.17${}^{a}$	1.03 ± 0.16${}^{a}$	1.01 ± 0.17	0.96 ± 0.24	0.03	0.16	0.21
Total fat content, kg	0.15 ± 0.05${}^{b}$	0.41 ± 0.11${}^{a}$	0.35 ± 0.12${}^{a}$	0.32 ± 0.13	0.32 ± 0.15	< 0.01	0.38	0.16
Total bone content, kg	0.44 ± 0.10	0.49 ± 0.08	0.47 ± 0.09	0.48 ± 0.07	0.45 ± 0.10	0.60	0.33	0.82
Total muscle content, %	49.38 ± 3.49	48.30 ± 2.38	48.22 ± 2.15	48.69 ± 2.74	48.41 ± 2.54	0.79	0.73	0.53
Total fat content, %	10.18 ± 2.56${}^{b}$	18.33 ± 3.52${}^{a}$	16.09 ± 3.25${}^{a}$	15.17 ± 4.48	15.50 ± 4.69	< 0.01	0.59	0.31
Total bone content, %	28.34 ± 3.26${}^{a}$	22.18 ± 2.51${}^{b}$	22.21 ± 4.41${}^{b}$	23.47 ± 2.64	23.81 ± 5.53	0.01	0.23	0.07
Muscle–fat ratio	5.08 ± 1.06${}^{a}$	2.74 ± 0.63${}^{b}$	3.14 ± 0.80${}^{b}$	3.54 ± 1.23	3.47 ± 1.31	< 0.01	0.45	0.65
Muscle–bone ratio	1.75 ± 0.31	2.21 ± 0.31	2.31 ± 0.81	2.10 ± 0.29	2.19 ± 0.79	0.03	0.13	0.15

Different lower-case letters in the same line indicate significant differences (p<0.05) between concentrate supplementation. Different upper-case letters in the same line indicate significant differences (p<0.05) between tropical pasture.

**Table 6:** Discriminant multivariate analysis with percentage of correct and error rate assigned to different concentrate supplementation and two types of tropical grass forage, using all variables, variables measured in kilograms, variables related to percentages, subjective and objective variables, variables related to adipose tissue, and variables related to muscularity and compacity of the carcass.

Measurements		Concentre supplementation			Tropical pasture	
		0 %	1.5 %	3 %	Aruana	Marandu
All variables	Correct, %	100.00	100.00	100.00	100.00	100.00
	Error rate	0.00	0.00	0.00	0.00	0.00
Weight in kilograms	Correct, %	100.00	100.00	100.00	88.86	80.00
	Error rate	0.00	0.00	0.00	0.11	0.20
Percentage	Correct, %	100.00	91.67	75.00	61.10	80.00
	Error rate	0.00	0.08	0.25	0.38	0.20
Subjective and objective	Correct, %	100.00	83.33	91.67	83.33	86.67
	Error rate	0.00	0.16	0.08	0.16	0.13
Indicators of adipose tissue	Correct, %	100.00	75.00	66.67	66.67	66.67
	Error rate	0.00	0.25	0.33	0.33	0.33
Indexes of muscularity and compacity	Correct, %	66.67	83.33	91.67	61.10	53.33
	Error rate	0.33	0.16	0.08	0.38	0.46

Joint analysis of the 29 variables revealed the heterogeneity of the carcasses depending on the dietary treatment used (Table 6). The use of all variables simultaneously generated no misclassifications when assigning a carcass to the supplementation of concentrate rates and pasture species (Table 6). The 16 quantitative variables expressed in weight in kilograms (Table 2) had perfect (100 %) discrimination of the carcasses, considering the concentrate rates, and discrimination of 88.6 %, considering the pasture species (Table 6). Three carcasses of lambs fed on Marandu grass were misclassified as fed on Aruana grass.

Eleven variables, in percentages (Table 2), had a strong differentiation between carcasses considering the concentrate rates (100.0 %, 91.7 %, and 75.0 % for 0.0 %, 1.5 %, and 3.0 % supplementation; Table 6). This subset (percentage) did not discriminate carcasses of animals fed on Aruana grass (61.1 %) but discriminated those fed on Marandu grass (80.0 %; Table 6).

The indices and descriptors of muscle, compacity, texture, quality, colour, and body score of the carcass (Table 2), discriminated carcasses (100.0 %, 83.33 %, and 91.67 %) considering the concentrate supplementation (0.0 %, 1.5 %, and 3.0 %, respectively; Table 6). These eight variables of objective and subjective measurements discriminated carcasses of animals fed on Aruana (83.3 %) and Marandu (86.7 %) grass.

The indicators of adipose tissue (n=6; Table 2) perfectly discriminated (100 %) carcasses of animals on grass-only diets (0.0 % concentrate). The variables related to adipose tissue had a poor discrimination (66.7 %) regarding the lambs with 3.0 % supplementation and lambs fed on different tropical pasture species (Aruana and Marandu).

The variables related to carcass muscularity and compacity indices (n=3; Table 2) discriminated carcasses of animals on diets with 1.5 % and 3.0 % concentrate (83.3 and 91.6 %, respectively; Table 6). Contrastingly, this subset did not discriminate carcasses of lambs with grass-only diets (66.7 %) or considering the pasture species (61.1 % and 53.3 % for Aruana and Marandu, respectively; Table 6).

In general, four subsets perfectly discriminated (100.0 %) the carcasses of animals fed on grass-only diets (0.0 % concentrate; Table 6). We found only slight differences between carcasses of animals that received concentrate levels of 1.5 % and 3.0 %. Regarding the pasture species, carcass was consistently different in the subsets from weight in kilograms, percentage, or objective and subjective measurements.

## 4 Discussion

Carcass weight, yield, muscularity, and adiposity were greater in animals on diets with concentrate levels of 1.5 % and 3 %. This was probably due to the greater levels of energy and protein in their diets (Carrasco et al., 2009b; Papi et al., 2011; Fernandes et al., 2011), which also can be a result of associative effect between forage and concentrate supplementation (Silva et al., 2011; Fajardo et al., 2015). Concentrate supplementation probably improved the metabolic efficiency and promoted faster digestion and lower gastrointestinal content at slaughter (Joy et al., 2008; Jacques et al., 2011; Armero and Falagán, 2015). The concentrate may have shortened the grazing time due to the better nutritional status of the animals (Silveira et al., 2015; Fajardo et al., 2015). This is related to higher resting intervals, which may explain their superior carcass mean values. The relation between concentrate supplementation muscularity and adiposity of the carcass has also been reported previously (Fernandes et al., 2011; Majdoub-Mathlouthi et al., 2013; Armero and Falagán, 2015). Finished lambs in grazing systems without supplementation results in lower fat accumulation in the carcass (Jacques et al., 2011; Papi et al., 2011; Majdoub-Mathlouthi et al., 2013; Armero and Falagán, 2015; Asadollahi et al., 2017).

The results of the CCI (Table 3) were consistent with those reported by other authors who evaluated tropical pastures (Carrasco et al., 2009a; Armero and Falagán, 2015). These indices may enable an objective evaluation of carcass conformation, indicating the connections between muscle mass, adipose mass, and bone length and determining the amount of tissue accumulated per length unit (Costa et al., 2019). Heavier carcasses (Table 3) resulted in heavier commercial cuts; this result is consistent with those found in a previous study (Rocha et al., 2018). Considering the leg composition and muscularity as indicators of carcass composition (Díaz et al., 2006), the results were similar to those found for the commercial cuts.

Regarding the total muscle content, previous studies (Carrasco et al., 2009a) have also reported that the total muscle content, measured in kilograms, varies according to the concentrate rates offered to lambs slaughtered at similar ages and weights; however, this is not the case for the TMC measured as a proportion (%).

The use of concentrate supplementation (1.5 % and 3.0 %) increased the TFC in the carcasses (kg and %; Table 5); this has also been reported previously (Armero and Falagán, 2015). A higher nutrient intake is probably connected to a better starch digestion, increased ruminal propionate production, higher insulin secretion, and improved fat synthesis (Bines and Hart, 1992).

Concentrate supplementation reduced the TBC as percentage, most likely because of the early maturation of bone tissue and the smaller proportion of bones at higher body weights (Díaz et al., 2006). Therefore, a poor diet may be connected to lower weights at slaughter and higher bone proportions in the commercial cuts.

Higher muscle–fat ratios in carcasses of lambs fed on grass-only diets (Table 5) have also been reported previously (Joy et al., 2008), mainly for animals in grazing systems with concentrate supplementation (Murphy et al., 1994; Borton et al., 2005).

Lambs from European breeds, similar to the animals used in this study, when slaughtered with 32 kg presented, in general, have great bone maturity (75 %) and a high potential to develop muscle (50 %) and fat (33 %) tissues (Rouse et al., 1970; Owens et al., 1993; Borton et al., 2005). Thus, the adoption of concentrate supplementation as a strategy towards the production of lambs for early slaughtering is a viable management strategy, and it is variable according the grass forage used.

However, the pasture species had low effects on the results of the analysis of variance (Tables 3, 5, and 6) but an obvious effect when all variables were considered in the multivariate approach (Table 6). This result must be interpreted carefully, because the number of phenotypical variables (n=29) was similar to that of the carcasses evaluated (n=36), which may have affected the stability of the linear discriminant function, leading to bias. Therefore, an exploratory evaluation may probably be more consistent using subsets of variables.

Using subsets of variables in percentage did not discriminate carcasses of animals fed on Aruana grass but discriminated carcasses of animals fed on Marandu grass (Table 6). This denotes that animals fed on Marandu grass generate a peculiar carcass phenotype, which is more evident on grass-only diets. Such a trend was not observed with Aruana grass (Table 6), most likely because of the greater oscillation of the nutritional composition of Aruana grass when compared to Marandu grass over short periods (Emerenciano Neto et al., 2014).

The results suggest that the quality of the phenotypic variable may be more relevant than the number of variables used in accessing and exploring phenotypic variability. As observed in some subsets, e.g. variables related to fat tissue (Table 6), the discrimination of carcasses of animals fed on diets with concentrate at rates of 1.5 % or 3.0 % was not consistent. Differentiation between carcasses was usually easier when comparing animals on grass-only diets. In fact, the concentrate rates for small ruminants raised in tropical grazing systems have not been established yet. However, according to the results of the multivariate analysis, concentrate supplementation was probably the determining factor for a better fat finishing of the carcass. These results were similar to those of the analysis of variance (Tables 3, 5, and 6) and consistent with previous reports (Dian et al., 2007; Carrasco et al., 2009a).

Only three variables related to carcass muscularity and compacity indices (Tables 2 and 6) discriminated carcasses, considering the concentrate rates of 1.5 % and 3.0 %. These indices are strongly connected to an increased protein and energy supply in the diet (Majdoub-Mathlouthi et al., 2013).

Distinct subsets of variables explained different effects on carcass variability and characteristics. Therefore, the effect of concentrate rates and pasture species on different geographic locations, genetic groups (breeds and interracial crossing), and different production systems should be disentangled to identify the phenotypic diversity of lamb carcasses in tropical regions.

## 5 Conclusions

The use of concentrate supplementation on rearing lambs improves the quality of carcass traits. The period of finishing can be shorted with supplementation of 3 % of body weigh in Aruana and Marandu grass. The discriminant analysis can identify the differences between rearing systems based on all carcass traits. This analysis can be used to develop carcass traceability systems.

## Data Availability

Data are available at (Vega Britez, 2016).
